# Severe pneumonia caused by *Chlamydia psittaci* and *Aspergillus terreus*: a case report

**DOI:** 10.3389/fmed.2026.1750879

**Published:** 2026-02-24

**Authors:** Chuanli Zhang, Jin Wang

**Affiliations:** 1 Department of Respiratory and Critical Care Medicine, Nanjing Jiangbei Hospital, Nanjing, China; 2 Department of Rehabilitation Medicine, Nanjing Jiangbei Hospital, Nanjing, China

**Keywords:** *Aspergillus terreus*, case report, *Chlamydia psittaci*, severe pneumonia, targeted next-generation sequencing

## Abstract

*Chlamydia psittaci* is a known cause of severe pneumonia and may be associated with invasive fungal co-infections; however, its complication by *Aspergillus terreus* is exceptionally rare. We report a case of a previously healthy 58-year-old woman with poultry exposure who presented with fever and dyspnea that rapidly progressed to respiratory failure requiring mechanical ventilation. After initial empirical antibiotics failed, targeted next-generation sequencing (tNGS) of bronchoalveolar lavage fluid concurrently identified *Chlamydia psittaci* and *Aspergillus terreus*, guiding successful targeted therapy with doxycycline and voriconazole, which led to full recovery. This case highlights the utility of tNGS in enabling rapid, simultaneous pathogen identification and guiding targeted therapy in severe pneumonia.

## 1 Introduction

Psittacosis, or “parrot fever,” is an acute zoonotic disease caused by *Chlamydia psittaci* (1). It is an important yet frequently overlooked cause of community-acquired pneumonia (CAP). Its non-specific clinical presentation often leads to delayed diagnosis and inappropriate initial treatment (2). Severe cases can rapidly progress to acute respiratory distress syndrome (ARDS) and respiratory failure (3). Recently, the clinical application of next-generation sequencing (NGS) has significantly improved the detection of *Chlamydia psittaci* pneumonia (4).

*Aspergillus terreus*, a conditionally pathogenic fungus within the *Aspergillus* genus, is widespread in soil and decaying organic matter (5, 6). It is a cause of invasive aspergillosis (IA) with a high mortality rate, particularly threatening immunocompromised hosts. *Aspergillus terreus* is clinically distinguished from other *Aspergillus* species by its intrinsic resistance to amphotericin B (AmB), a cornerstone polyene antifungal. This key phenotypic difference necessitates a distinct therapeutic strategy, making voriconazole the recommended first-line agent for *Aspergillus terreus* infections per Infectious Diseases Society of America (IDSA) guidelines (7).

Retrospective studies indicate that fungal co-infections are not uncommon in severe psittacosis, with *Aspergillus* species being among the most frequently reported pathogens (8–10). However, co-infection with *Aspergillus terreus* is extremely rare. We report a case of severe pneumonia due to co-infection with *Chlamydia psittaci* and *Aspergillus terreus* in a previously immunocompetent woman, accurately diagnosed via tNGS and successfully treated. This case provides a diagnostic and therapeutic framework for managing complex mixed infections using advanced diagnostics and targeted antimicrobial therapy.

## 2 Case description

A 58-year-old woman with no prior medical history was admitted on October 7, 2025. Five days before admission, she developed fever and vomiting. Two days of home rest were ineffective, so she received intravenous cephalosporins and antiemetics for 2 days at a local hospital. During this treatment, her condition failed to improve and she developed dyspnea and a mild cough, leading to her transfer and admission. Epidemiological inquiry revealed daily exposure to domestic poultry.

**Figure 1:**
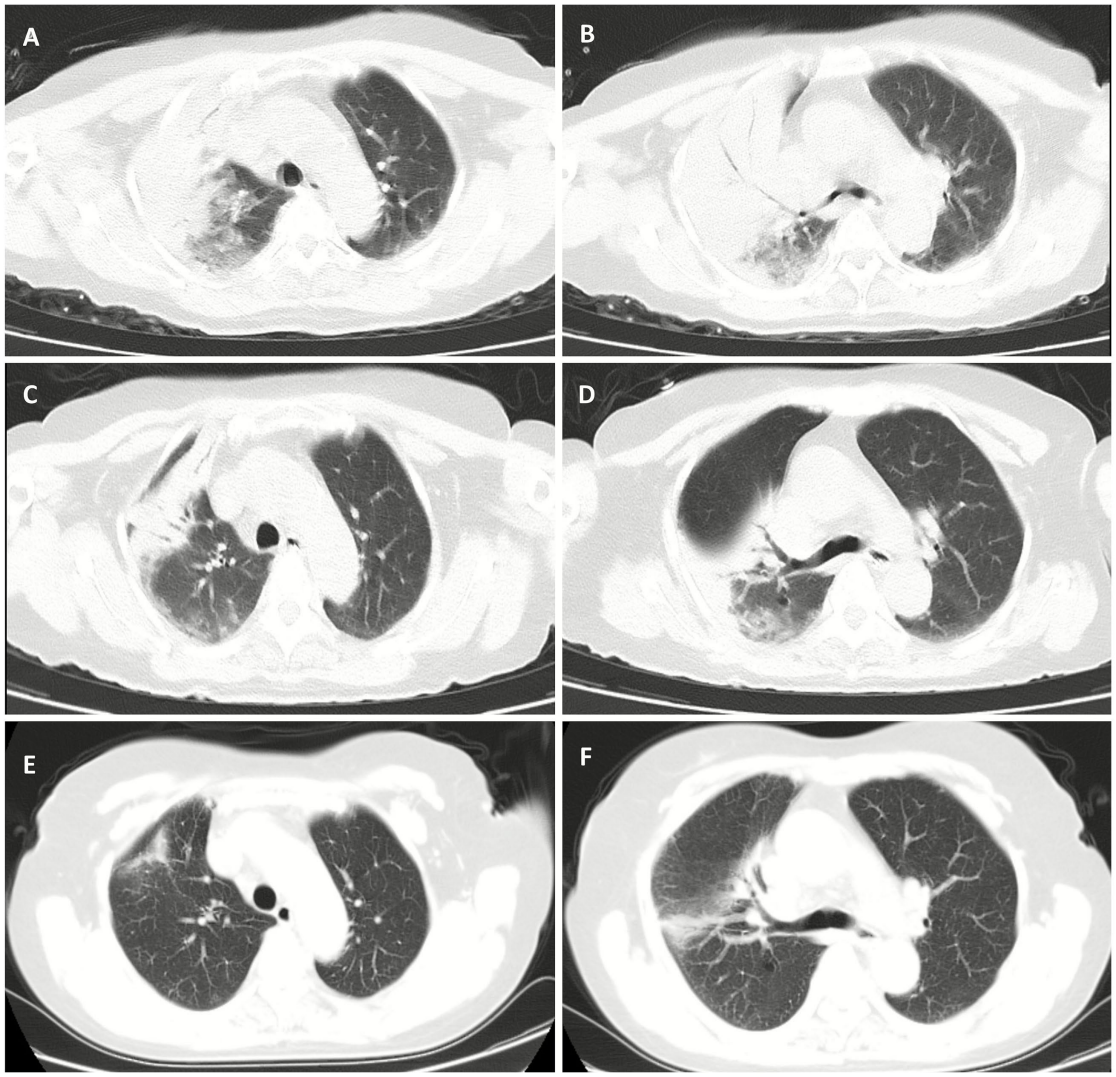
Serial chest CT images of the patient during hospitalization and follow-up. **(A,B)** At admission (day 1). **(C,D)** Day 15 of hospitalization. **(E,F)** 9 days after discharge.

On admission, her temperature was 37.7°C, heart rate 110 beats/min, respiratory rate 30 breaths/min, and blood pressure 167/86 mmHg. Lung auscultation revealed moist and dry rales in the right lung. Laboratory tests on admission (Table 1) revealed leukocytosis with neutrophilia, severe lymphopenia, and elevated hepatic enzymes, creatine kinase, and lactate dehydrogenase. Inflammatory markers (C-reactive protein [CRP], procalcitonin [PCT], Interleukin-6 [IL-6], and heparin-binding protein [HBP]) and D-dimer were significantly increased. Arterial blood gas under an oxygen mask (10 L/min) showed pH 7.50, PaO₂ 87 mmHg, and PaCO₂ 25 mmHg. Doppler ultrasound of the lower extremities confirmed a thrombus in the left calf muscular vein. Chest CT revealed consolidation in the right lung and a small pleural effusion (Figures 1A,B).

**Table 1:** Laboratory test results.

Laboratory test	Result	Reference interval
White blood cell (×10^9^/L)	13.4	3.5–9.5
Neutrophil ratio (%)	95.7	40.0–75.0
Lymphocyte (×10^9^/L)	0.31	1.10–3.20
Procalcitonin (ng/mL)	4.91	0.00–0.05
C-reactive protein (mg/L)	260.8	0.0–10.0
CD4 + T cell (%)	18.77	20.98–56.22
CD8 + T cell (%)	47.62	51.06–88.24
Heparin-binding protein (ng/mL)	>300.00	≤11.40
Interleukin-6 (pg/mL)	630.46	≤20.00
Interleukin-8 (pg/mL)	73.21	≤21.40
Interleukin-10 (pg/mL)	17.84	≤5.90
Alanine Aminotransferase (U/L)	72	0–34
Aspartate Aminotransferase (U/L)	108	15–46
γ-Glutamyl transferase (U/L)	93	12–43
Alkaline phosphatase (U/L)	108	38–126
Creatine kinase (U/L)	1,051	30–135
Lactate dehydrogenase (U/L)	425	100–246
D-Dimer (μg/mL)	9.55	0.00–1.00
BALF galactomannan assay	0.56	≤0.8
Routine sputum culture for bacteria	Negative	Negative
Blood culture	Negative	Negative

The patient was diagnosed with severe community-acquired pneumonia. She was treated with empirical broad-spectrum antibiotics (meropenem 1.0 g ivgtt q6h and levofloxacin 0.5 g ivgtt qd) along with supportive care such as bronchodilators and anticoagulation. However, she remained febrile with a temperature spiking to 39.6°C, and with persistent dyspnea.

On hospital day 3, her respiratory status worsened acutely and was accompanied by mental status changes. Repeat blood gas (on 10 L/min oxygen via face mask) showed pH 7.48, PaO₂ 67 mmHg, and PaCO₂ 29 mmHg, prompting endotracheal intubation and mechanical ventilation. Given her exposure history and clinical course, psittacosis was strongly suspected. Levofloxacin was replaced with oral doxycycline (0.1 g po bid). Bronchoscopy with bronchoalveolar lavage (BAL) was performed, and bronchoalveolar lavage fluid (BALF) was sent for microbiological culture and tNGS.

Microbiological analysis was performed as follows. For tNGS, a commercial assay (Genoxor Medical & Technology) was employed. Libraries were prepared via hybridization capture and sequenced on an MGISEQ-200 platform. Bioinformatic analysis included host sequence depletion, alignment using the Burrows-Wheeler Aligner (BWA), and application of positivity thresholds (≥10 unique reads, mismatch rate <10%). Semi-quantitative concentrations (copies/mL) were calculated based on internal calibration standards, with a turnaround time of approximately 48 h. In parallel, BALF was cultured on Columbia blood agar and chocolate agar at 35°C for 5 days, and fungal isolates were identified by matrix-assisted laser desorption/ionization time-of-flight mass spectrometry (MALDI-TOF MS).

On hospital day 6, tNGS detected *Chlamydia psittaci* (5.07 × 10^3^ copies/mL) and *Aspergillus terreus* (3.0 × 10^1^ copies/mL) (Table 2). Subsequent BALF cultures consistently grew *Aspergillus terreus*, confirming the co-infection. Voriconazole (200 mg po bid) was added. Antifungal susceptibility testing and therapeutic drug monitoring were not performed during treatment.

**Table 2:** Targeted next-generation sequencing of BALF^a^.

	Microorganisms	Estimated concentration(copies/mL)
List of detected pathogens	*Chlamydia psittaci*	5.07 × 10^3^
	*Aspergillus terreus*	3.0 × 10^1^

a The pathogen concentration is a semi-quantitative estimate based on the tNGS assay’s internal calibration.

**Figure 2:**
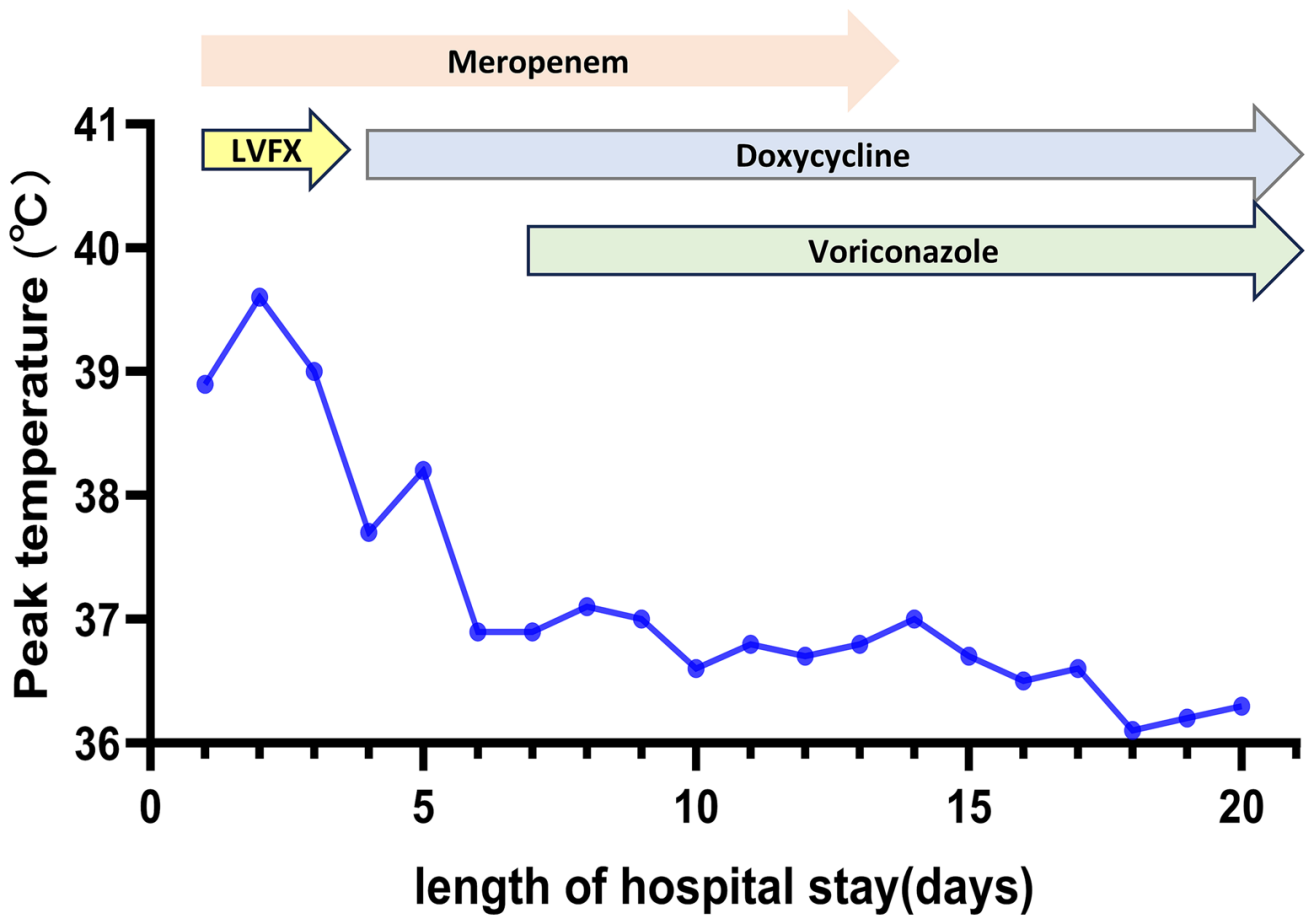
Maximum body temperature and antibiotic treatment during hospitalization. LVFX, levofloxacin.

**Figure 3:**
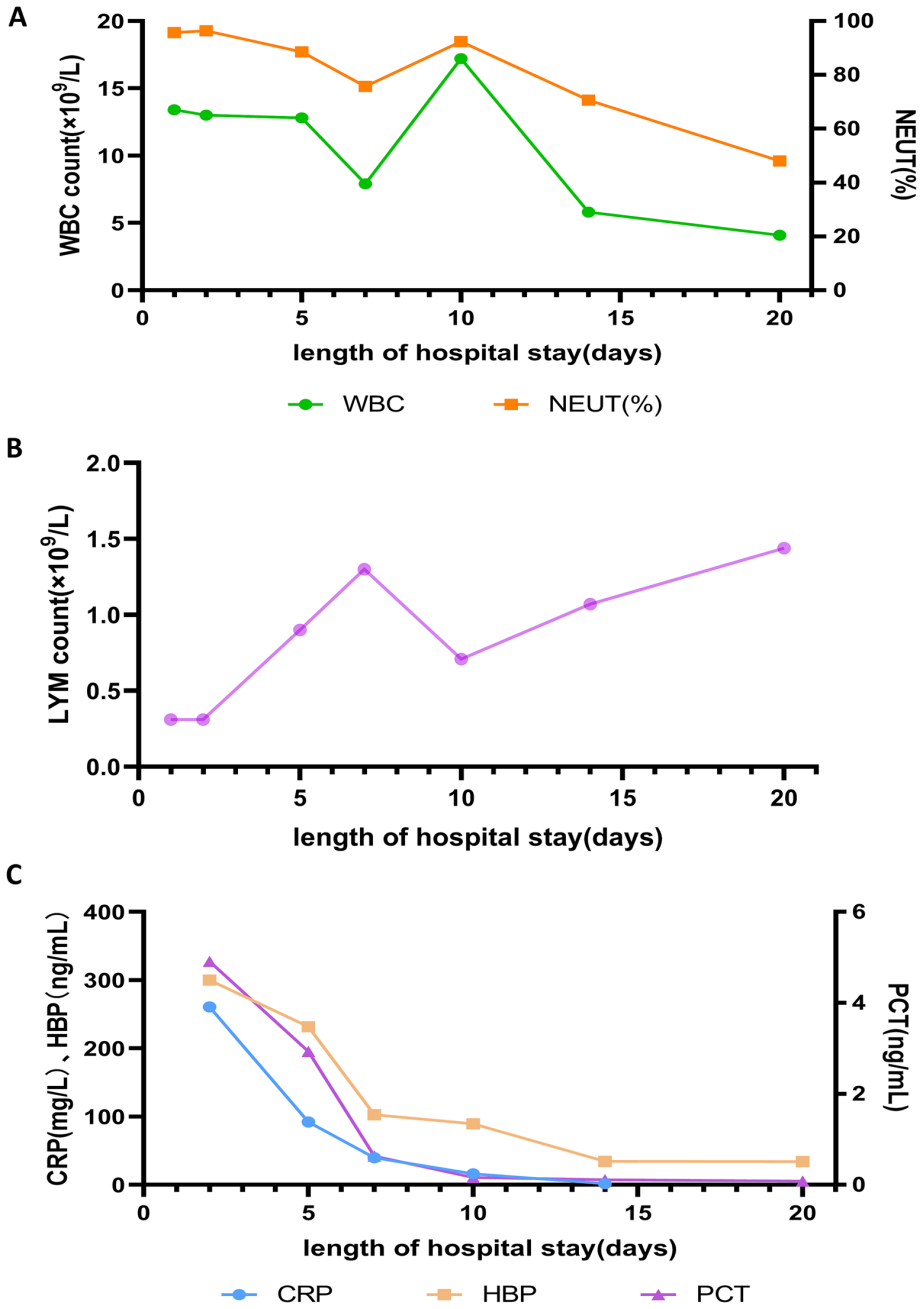
Changes in **(A)** white blood cell (WBC) count and neutrophil percentage (NEUT%), **(B)** lymphocyte (LYM) count, and **(C)** PCT, CRP, and HBP during hospitalization.

**Figure 4:**
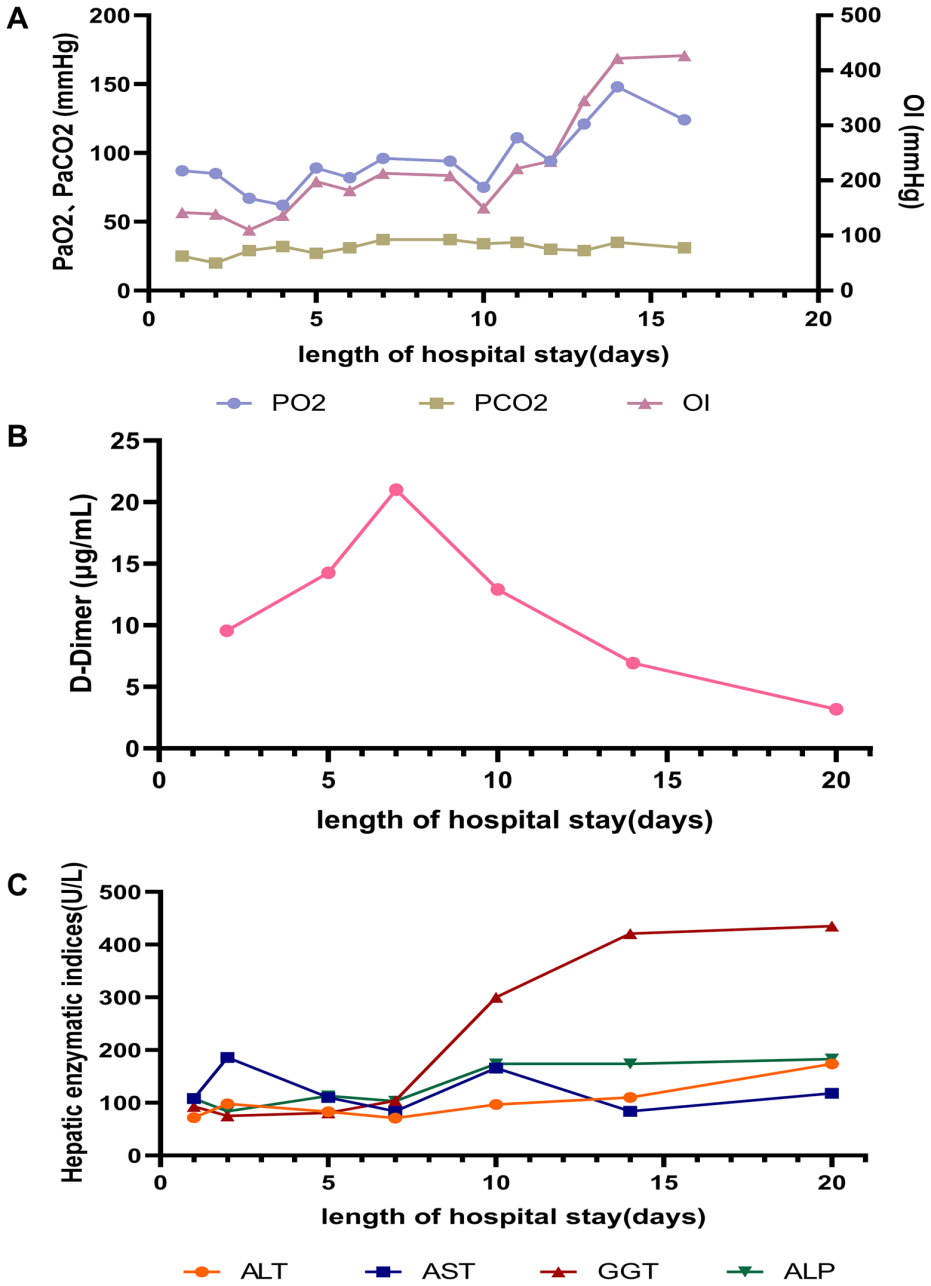
Changes in laboratory parameters during hospitalization: **(A)** Lung; **(B)** Coagulation function; **(C)** Liver. OI, Oxygenation index (PaO_2_/FiO_2_).

Following targeted treatment, the patient’s condition improved steadily, marked by the resolution of fever (Figure 2), improved oxygenation, and a general favorable trend in laboratory parameters (Figures 3, 4). On hospital day 8, she was successfully weaned from mechanical ventilation and transitioned to high-flow nasal oxygen. A transient elevation in white blood cells and concomitant lymphopenia observed the following day (October 16) were considered related to steroid administration at the time of extubation. A follow-up chest CT on day 15 showed significant resolution of pulmonary consolidation (Figures 1C,D), prompting the discontinuation of meropenem. The patient was discharged on hospital day 21 on a regimen of oral voriconazole, doxycycline, liver-protective agents, and rivaroxaban. Imaging at the 9-day follow-up after discharge revealed near-complete resolution of the pulmonary lesions (Figures 1E,F).

## 3 Discussion

To our knowledge, this is the first reported case of severe *Chlamydia psittaci* pneumonia co-infected with *Aspergillus terreus* that was successfully managed. This case expands the clinical spectrum of psittacosis. It demonstrates that the infection can cause not only severe bacterial pneumonia but may also be associated with a state of critical illness-related immunosuppression. Psittacosis often presents non-specifically and can be mistaken for other CAP types (11). As in our case, a history of avian exposure is a critical epidemiological clue, though it may be overlooked without deliberate inquiry. In patients with severe CAP unresponsive to empirical antibiotics, psittacosis and other rare pathogens should be considered.

Standard diagnostic guidelines for invasive aspergillosis recommend a work-up including serum and BALF galactomannan (GM) assays and chest CT imaging for characteristic signs (e.g., halo sign) (7). In our patient, BALF GM assay was negative, and chest CT showed extensive consolidation without typical fungal features. These findings are not uncommon in invasive aspergillosis, particularly in non-neutropenic patients or those with concurrent bacterial pneumonia. In this context, tNGS of BALF proved to be a pivotal, culture-independent tool for rapid, unbiased pathogen identification (12). It provided a definitive diagnosis within 48 h, concurrently identifying *Chlamydia psittaci* and *Aspergillus terreus*.

Accurate differentiation between *Aspergillus* species was critical. Although *Aspergillus fumigatus* is the most common cause of invasive aspergillosis, *Aspergillus terreus* was identified by tNGS and confirmed by mycological culture. This culture-based confirmation validated the molecular result and was clinically decisive, directly guiding the guideline-adherent choice of voriconazole over AmB (7). The combination of doxycycline and voriconazole created a synergistic regimen that effectively targeted both pathogens.

The mechanisms by which *Chlamydia psittaci* infection predisposes to invasive aspergillosis warrant further study. Similar to other severe infections, a potent systemic inflammatory response, characterized by elevated IL-6 and CRP, can induce endothelial injury and a pro-thrombotic state (13). Our patient exhibited laboratory (elevated D-dimer) and clinical (left calf muscular vein thrombosis) evidence of this. Therapeutic anticoagulation with rivaroxaban was initiated, likely preventing thrombotic extension, although its direct impact on the infectious course is uncertain.

More importantly, this inflammatory milieu, coupled with direct cellular damage, may disrupt pulmonary mucosal immunity and epithelial integrity, creating a portal for filamentous fungi (14). A key feature of this case is the profound, persistent lymphopenia and notably low CD4 + T-cell count, which may indicate a state of transient, infection-associated immunosuppression. This observed immunosuppression likely served as the pivotal factor facilitating invasion by this opportunistic fungus. This case highlights the possibility that dysregulated inflammation in psittacosis leads not only to coagulopathy but also to immunosuppression, potentially contributing to life-threatening fungal co-infections. This highlights the importance of monitoring immune cell subsets in severe pneumonia to stratify the risk of secondary infections.

In conclusion, we describe a rare, life-threatening co-infection with *Chlamydia psittaci* and *Aspergillus terreus*, complicated by a parainfectious thrombotic event. In patients with severe CAP refractory to standard antibiotics, particularly those with avian exposure and profound lymphopenia, clinicians should suspect psittacosis and possible secondary fungal invasion. Empirical anti-psittacosis therapy should be initiated early, and advanced diagnostics like tNGS employed aggressively. Vigilance for both thrombotic complications and signs of immunosuppression is essential. This case demonstrates that precise microbiological diagnosis combined with targeted antimicrobial and adjunctive therapy (including anticoagulation) can achieve favorable outcomes even in severe, complex co-infections.

## 4 Patient perspective

I used to think of myself as a healthy person. But in early October, what started as a simple fever and upset stomach quickly spiraled into the most frightening experience of my life. Within days, I could not catch my breath. It felt like I was drowning on dry land. I was rushed to the hospital, and my memories of the first few days are a blur of tubes, machines, and the constant sound of alarms.

The scariest moment was when the doctors told me they needed to put me on a breathing machine. I was terrified. I remember thinking about my family and wondering if I would ever see them again. The initial medicines were not working, and no one knew exactly what was wrong with me. It felt like a race against time. The turning point came when the doctors did a lung wash and used a new kind of test. For the first time, they could tell me exactly what I was fighting: not one, but two rare infections, something called “parrot fever” from my chickens at home and a fungal infection. Finally, we had answers. They switched my medicines to target these specific germs. It was a slow and difficult journey, but day by day, I could feel myself getting stronger. The day I was able to breathe on my own again was the day I knew I was going to make it.

I am so grateful to the medical team who never gave up on me. Their expertise and the power of that advanced test saved my life. My advice to others is to never ignore a persistent fever, especially if you have animals. And if you get sick and the usual treatments aren’t working, ask your doctors to keep looking. A precise diagnosis can make all the difference between life and death. Today, I am back home and feeling well, but I will never forget how fragile life can be.

## Data Availability

The original contributions presented in the study are included in the article/supplementary material, further inquiries can be directed to the corresponding author.
